# Correlations between exploratory eye movement, hallucination, and cortical gray matter volume in people with schizophrenia

**DOI:** 10.1186/s12888-018-1806-8

**Published:** 2018-07-13

**Authors:** Linlin Qiu, Hao Yan, Risheng Zhu, Jun Yan, Huishu Yuan, Yonghua Han, Weihua Yue, Lin Tian, Dai Zhang

**Affiliations:** 1 Department of Medical Psychology, Chaohu Hospital, Anhui Medical University, Hefei, Anhui China; 2 Anhui Province Key Laboratory of Cognition and Neuropsychiatric Disorders & Collaborative Innovation Center of Neuropsychiatric Disorders and Mental Health, Hefei, Anhui China; 3 Peking University Sixth Hospital (Institute of Mental Health), Beijing, China; 4 National Clinical Research Center for Mental Disorders & Key Laboratory of Mental Health, Ministry of Health (Peking University), Beijing, China; 5 The Department of Radiology, Peking University Third Hospital, Beijing, China; 6 Department of Psychiatry, the Affiliated Wuxi Mental Health Center of Nanjing Medical University, Wuxi, Jiangsu China

**Keywords:** Schizophrenia, Exploratory eye movement, Hallucination, Positive and negative syndrome scale, Gray matter volume

## Abstract

**Background:**

Widespread cortical gray matter alternations in people with schizophrenia are correlated with both psychotic symptoms and cognitive/behavioral abnormalities, including the impairments of exploratory eye movement (EEM). Particularly, the loss of gray matter density is specifically related to deficits of the responsive search score (RSS) of EEM in schizophrenia. It is unknown, however, whether the schizophrenia-related RSS deficits are associated with certain psychotic symptoms, such as hallucinations.

**Methods:**

In 33 participants with schizophrenia, the measurement of EEM, assessment of the hallucination severity using Positive and Negative Syndrome Scale (PANSS) and a voxel-based morphometric analysis of cortical gray matter volume (GMV) were conducted to investigate the relationships between the RSS of EEM, symptom severity, and GMV. In 29 matched healthy controls, the measurement of EEM and a voxel-based morphometric analysis of cortical GMV were also conducted to investigate the relationship between the RSS of EEM and GMV.

**Results:**

In participants with schizophrenia, the hallucination severity was significantly negatively correlated with both the RSS and the GMV of a large number of brain regions in the frontal, temporal, parietal, orbitofrontal, calcarine, cingulate, and insular cortices, and rolandic operculum, hippocampus, parahippocampal gyrus, and thalamus. Also in participants with schizophrenia, the RSS was significantly positively correlated with the GMV in the left supplementary motor area (SMA), left superior frontal cortex (SFG), bilateral precentral gyri, bilateral postcentral gyri, and bilateral middle frontal cortices. More importantly, the GMV of the SMA, SFG, and precentral gyrus in the left hemisphere was not only significantly negatively correlated with the hallucination severity but also significantly positively correlated with the RSS. No significant correlation could be revealed between the RSS and the GMV of any brain regions in healthy controls.

**Conclusions:**

There was a significantly negative association between the hallucination severity and the RSS of EEM, suggesting that the RSS may be a potential biomarker for predicting the hallucination severity of schizophrenia. Also, the GMV of the left SMA, SFG, and precentral gyrus may be the common substrates underlying both hallucination induction and the RSS in people with schizophrenia.

**Electronic supplementary material:**

The online version of this article (10.1186/s12888-018-1806-8) contains supplementary material, which is available to authorized users.

## Background

Hallucinations have been considered as critical characteristics in people with schizophrenia [1]. It has been suggested that investigation of the relationship between deficits of perceptual/cognitive processes and typical symptoms (such as hallucinations) of schizophrenia is important for understanding the nature of this disorder [2–4]. People with schizophrenia steadily display impairments in various cognitive domains, including perception, attention, learning, memory, inhibitory control, imagery and representation, executive function (motor control), language, general intelligence, and social communication [1, 5]. Particularly, the eye movement abnormality is one of the most reliable and reproducible impairments associated with schizophrenia [6–18]. Although eye movements are also vulnerable to pathological changes underlying many other neurodegenerative diseases [19–23], the impairment of exploratory eye movement (EEM) appears to be more specific to schizophrenia [12, 24–27]. For example, in an EEM task, compared to healthy controls, people with schizophrenia exhibit fewer eye fixations, longer mean duration of fixation, and shorter mean scanning length [12, 25, 28]. More in detail, EEM can be examined by measuring the participant’s eye tracking when a stationary S-shaped figure is viewed. In total 5 commonly used parameters can be obtained from eye tracking data analyses, including number of eye fixations (NEFs), total eye scanning length (TESL), mean eye scanning length (MESL), responsive search score (RSS), and cognitive search score. Among the 5 parameters, only the RSS (which measures the pattern of eye fixation points after the following question “Are there any other differences?” is asked) has been consistently reported to be vulnerable to schizophrenia across studies [7, 10, 12, 28].

Since the RSS is an integrated measure of the abilities for fine discrimination, selective and sustained attention, perception, working memory (including working memory of the S-shape figure), and execution (including imagery of the S-shape figure), any schizophrenia-related impairment of one or more of these abilities would reduce the RSS score. Takahashi et al. (2008) [10] have suggested that RSS impairment is an intermediate phenotype and a vulnerability marker for schizophrenia.

Convergent evidence has suggested that brain structural abnormality is one of the most critical pathological substrates underlying schizophrenia [29, 30]. Abnormal smooth pursuit and saccadic eye movements in people with either first-episode or chronic schizophrenia are associated with certain brain structural abnormalities [31–33]. The recent Qiu et al. study (2011) [12] has shown that both declined RSS and widespread gray-matter loss are observed in people with schizophrenia. Also, the RSS decline is significantly associated with the gray matter density (GMD) reduction in the occipito-tempro-frontal circuitry, which underlies both visual information and eye movement processing. Particularly, the findings of the Qiu et al. (2011) [12] study also support the views that RSS in people with schizophrenia is related to GMD in the right frontal eye field and right inferior frontal region [34], and the RSS impairment can be used as a phenotype and a vulnerability marker for schizophrenia [10].

Schizophrenia has potentially neurodevelopmental and neurodegenerative causes [35–38]. The neurodegeneration is likely the only one aspect of structural variability in people with schizophrenia. Schizophrenia-related brain structural damages are markedly correlated with not only cognitive function deficits but also positive psychotic symptoms [39]. More specifically, people with schizophrenia exhibit widespread reductions of both GMD [40, 41] and gray matter volume (GMV) [40, 42–45] in many brain regions, including the GMD reduction in the occipito-tempro-frontal circuitry, which underlies both visual information and eye movement processing [12]. It is noteworthy that GMD and GMV should not be treated as being tightly related (or equivalent) measures of regional gray matter integrity and may reflect different pathological processes [40, 46–48]. GMD is a scalar measure depending on image segmentation and signal intensity and reflects the proportion of gray matter within a given voxel after spatial normalization of the images; GMV reflects an estimate of absolute gray matter volume calculated by correcting for the deformations introduced during the spatial normalization procedure (a step termed “modulation”) [40]. Thus, GMD and GMV represent different gray-matter cytoarchitectural characteristics. The recent Gennatas et al. study (2017) [46] has shown that in young people with ages from 8 to 23 years, GMV generally decreases but GMD increases with age. Also, females have lower GMV but higher GMD than males throughout the brain. Thus, the Gennatas et al. study [46] has confirmed that GMD and GMV are not correlated measures for the gray matter quantity. Our previous study [12] has shown that the RSS is positively correlated with GMD in the brain regions that are disrupted in people with schizophrenia. However, whether the RSS is also positively correlated with GMV in certain brain regions in either people with schizophrenia or healthy people has not been reported in the literature.

It is widely accepted that GMV in certain cortical regions (including the frontal, temporal, and parietal cortices) is negatively correlated with hallucinations [44, 49–52]. Since the reduction of prefrontal cortical volume and hallucinations are also associated with the susceptibility gene for schizophrenia and schizoaffective disorder, DISC1 [49], studies of the relationship between behavioral/cognitive deficits and hallucinations of schizophrenia are critical for understanding the pathopsychological processes and the endophenotype of this mental disorder [3, 53, 54].

As mentioned above, the gray matter loss in people with schizophrenia is significantly related to the impairment of RSS [12], leading to that impairment of EEM can be used as a simplified biomarker of schizophrenia [55]. The RSS deficits in people with schizophrenia are significantly associated with GMD reductions in the occipital, temporal, and frontal regions underlying both visual information and eye movement processing [12, 34]. On the other hand, in people with schizophrenia the hallucination severity is also associated with the GMV alternations in the parietal, temporal, frontal, and paralimbic systems [39, 44, 49–52]. In addition to schizophrenia-related GMD, it is also of importance and interest to know whether the schizophrenia-related GMV are associated with the schizophrenia-related RSS. Besides, as mentioned above, the GMV in the frontal, temporal, and parietal cortices is negatively correlated with hallucinations [44, 49–52]. It is even more important to know whether the schizophrenia-related GMV are the common neural substance underlying both the RSS and hallucination in people with schizophrenia. However, up to date, there have been very few studies approaching these issues.

In our previous study [12], both declined RSS and widespread decreased GMD were observed in people with schizophrenia. In the present study, to further investigate whether the RSS is a potential biomarker of schizophrenia, the relationship between the RSS, hallucination severity, and GMV were examined in people with schizophrenia. In addition to EEM recordings, both measures of hallucination severity and voxel-based morphometric analyses of GMV were carried out to investigate (1) whether the RSS is correlated with the hallucination severity of schizophrenia; (2) whether the hallucination severity of schizophrenia is correlated with the GMV in certain cortical regions; (3) whether the RSS is correlated with the GMV in certain cortical regions; (4) more importantly, whether the GMV in some cortical regions is correlated with both the hallucination severity and the RSS of schizophrenia. Based on the existing literature, we hypothesize that the GMV in certain cortical areas may be negatively correlated with the hallucination severity but positively correlated with the RSS in people with schizophrenia.

## Methods

### Participants

Thirty-three right-handed people with schizophrenia, recruited from the Institute of Mental Health at Peking University, participated in this study. Twenty-nine age- and sex-matched right-handed healthy people were recruited from the communities near the institute as the healthy controls (HCs). All the HCs had no psychiatric histories or family histories of schizophrenia spectrum disorders. All the participants were the same ones that participated in our previous study [12], in which neither the hallucination severity nor the GMV was investigated. The clinical diagnosis was made by two independent psychiatrists using the ICD-10 diagnostic criteria for research for schizophrenia with paranoid subtype [56]. All people with schizophrenia received antipsychotic medications which were converted into chlorpromazine-equivalent dose [57–59]. A trained and experienced psychiatrist assessed the clinical symptoms (including hallucination severity) of these participants using the PANSS [60]. Magnetic resonance imaging (MRI) scans and EEM tasks were conducted on the same day. Exclusion criteria were epilepsy, mental retardation, severe physical disease, and treated with electroconvulsive therapy within the past 6 months. The right handedness of all the participants was verified by the Edinburgh Handedness Inventory [61]. All participants and patients’ legal guardians on behalf of the patients gave their written informed consent to participate in the study. Table 1 provides detailed demographic and clinical data. The study was approved by the Medical Research Ethics Committee of the Institute of Mental Health, Peking University.

**Table 1:** Demographic and clinical data of 33 participants with schizophrenia and 29 healthy controls

Subject characteristics	Participants with schizophrenia	Healthy controls	Statistics	*P* value
Male/female	19 / 14	17 / 12	χ^2^ = 0.01	0.93^a^
Age (years)	23.45 (4.05)^b^	23.17 (3.05)	*t* = 0.31	0.11^c^
Education (years)	13.67 (2.12)	14.38 (1.97)	z = − 1.38	0.17^d^
Intracranial volume (ml)	1647.35 (238.45)	1645.73 (182.27)	z = − 0.15	0.88^d^
Age at onset (years)	19.48 (3.37)	–		
Duration of illness (months)	41.27 (33.09)	–		
Antipsychotic dose (mg/day)^e^	437.27 (268.92)	–		
PANSS positive score	19.64 (4.49)	–		
PANSS negative score	15.94 (4.50)	–		
PANSS total score	67.79 (11.47)	–		

^a^Pearson Chi-square test

^b^Mean (standard deviation)

^c^Two sample t-test

^d^Mann-Whitney Test

^e^Chloropromazine-equivalent dose

PANSS Positive and Negative Syndrome Scale

### Procedures

#### EEM data acquisition and processing

The methods in this study were based on Kojima et al. (1992) [7]. The EEM examination was conducted in all the participants including 33 people with schizophrenia and 29 HCs using a nac 8-B type Eye Mark Recorder (nac, Tokyo, Japan). The procedure was briefly described as follows.

**Fig. 1:**
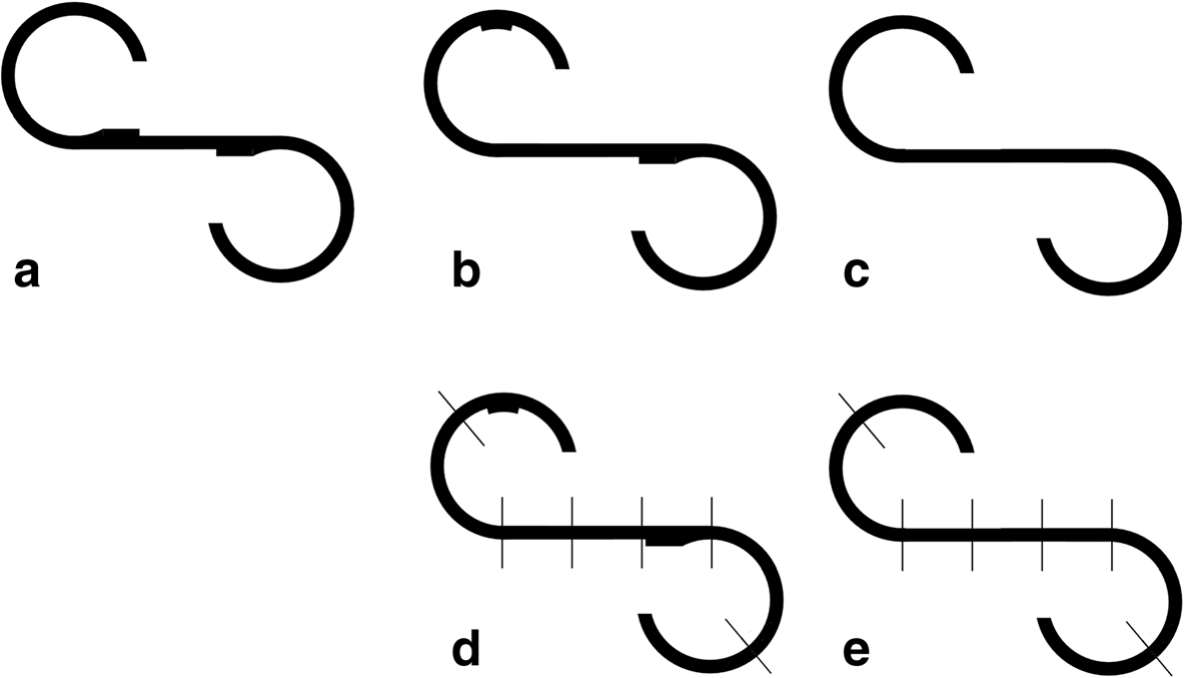
The original target figure (**a**) and two figures (**b**, **c**) that slightly differ from the target. Each of the two figures (**b**, **c**) is divided into seven sections (**d**, **e**)

The projections of three S-shaped figures were individually presented to the participants. First, each participant was shown the original target S-shaped figure (Fig. 1a) for 15 s and then immediately was instructed to draw the target figure from memory (a retention task). Second, the participant was shown the original target figure and two other slightly different figures (Fig. 1b, c) for 15 s. While each figure was being viewed, the participant was asked to make comparison and answer whether it differed from the original target figure, and if so, how it differed. Specifically, following the participant’s responses, while still viewing the figure, the participant was then asked, “Are there any other differences?” [This question was repeated until the participant stated that there were no differences (a comparison task)]. Third, the participant was asked to view the projection of the original target figure again for 15 s and to draw it again. The eye movements were recorded on a videotape and analyzed with a computerized system. The two slightly different figures (Fig. 1b, c) were each divided into seven sections (Fig. 1d, e) and the RSS (the number of sections on which the subjects fixed their eyes once or more in the completion of the comparison task) was calculated and analyzed [9].

#### MRI data acquisition and processing

Imaging data were acquired at the Department of the Radiology, the Third Hospital, Peking University, using a 3.0-Tesla Magnetom Trio MR system (Siemens Medical System, Erlangen, Germany). A 3D-MPRAGE sequence was employed to obtain high resolution three-dimensional T1-weighted images in a sagittal orientation with the following specifications: time repetition (TR) = 2350 ms, time echo (TE) = 3.44 ms, field of view (FOV) = 256 × 256 mm^2^, flip angle = 7°, matrix size = 256 × 256, 192 sagittal slices, slice thickness = 1 mm, total acquisition time = 363 s. Imaging data were analysed using the Statistical Parametric Mapping Software (SPM5, Welcome Department of Imaging Neuroscience, London; available at http://www.fil.ion.ucl.ac.uk/spm). In addition, the VBM5 toolbox http://dbm.neuro.uni-jena.de/vbm) was employed to perform a VBM analysis implementing segmentation algorithm from SPM5 and extending the core segmentation algorithm by adopting the Hidden Markov Random Field (HMRF) model [62]. In brief, after normalization, the segmented gray matter images were then modulated by calculating the Jacobian determinants, derived from the special normalization step, and multiplying each voxel by the relative change in volume, as in the method of Good et al. (2001) [63]. Resulting GMV images were smoothed with a 12 mm full width-half maximum (FWHM) Gaussian kernel.

#### Statistical analyses

Statistical analyses were carried out with SPSS for windows (SPSS 13.0, SPSS Inc., Chicago, IL, USA). To identify the relationship between the hallucination severity and RSS in participants with schizophrenia, Spearman’s correlation was performed. In addition, the relationship between RSS and the other positive and all the negative subscales on the PANSS were also examined using Spearman’s correlation. Statistical significance was set at *p* < 0.05 (two-tail, uncorrected for multiple comparison). Scatter plot showing the relationship between the hallucination severity and RSS was created using SPSS for windows.

The images were analyzed within the framework of the general linear model implemented in SPM5. First, to examine the relationship between the hallucination severity and GMV in participants with schizophrenia, a multiple regression model with hallucination as independent variable was performed, using age, gender, antipsychotic dose, duration of illness, and intracranial volume as confounding covariates. Second, to examine the relationship between RSS and GMV in participants with schizophrenia and HCs, respectively, multiple regression models with RSS as independent variable were used. The multiple regression model for participants with schizophrenia included age, gender, antipsychotic dose, duration of illness, and intracranial volume as confounding covariates; and the multiple regression model for HCs included age, gender, and intracranial volume as confounding covariates. Finally, to explore whether there was GMV of certain cortical regions correlated with both hallucination severity and RSS and in participants with schizophrenia, an overlapped map was generated by calculating the intersection of the two thresholded statistical parameter maps.

Considering that a significantly positive correlation between RSS and GMD in people with schizophrenia has been documented in the previous study [12], this study additionally examined the relationship between the hallucination severity and GMD in participants with schizophrenia. A multiple regression model with hallucination as independent variable was performed, using age, gender, antipsychotic dose, and duration of illness as confounding covariates.

A voxel-level of *p* < 0.05 (two-tailed) and the minimum cluster size estimated by the non-stationary correction in the VBM5 toolbox based on random field theory were utilized to correct for all the results of the MRI analyses [64, 65].

## Results

### Correlations between hallucination severity and RSS

**Fig. 2:**
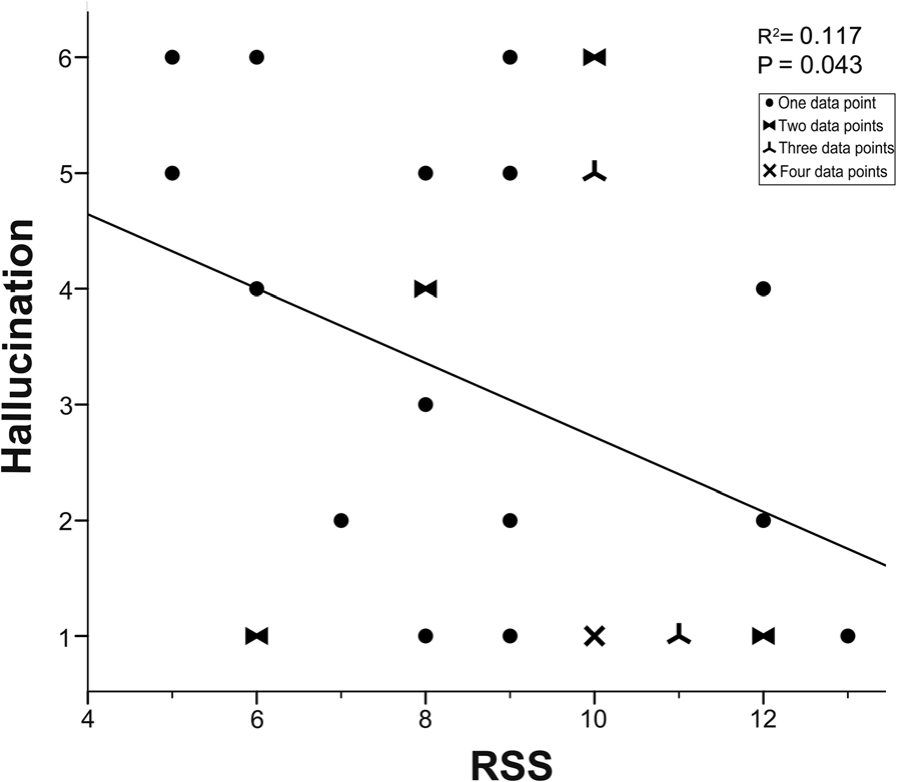
The Scatter plot showing the significantly negative correlations between the hallucination severity and the responsive search score (RSS) in 33 participants with schizophrenia. Data overlaps with two, three, and four individuals are indicated by different symbols, respectively

In 33 participants with schizophrenia, the hallucination score on the PANSS was between 1and 6 (mean = 3.00, standard deviation = 2.03) and the RSS value was between 5 and 13 (mean = 9.12, standard deviation = 2.16). The hallucination severity was significantly negatively correlated with the RSS (*r* = − 0.354, *p* = 0.043, uncorrected). The scatter plot exhibiting the relationship between the hallucination severity and the RSS is shown in Fig. 2.

In addition to the hallucination severity, the assessment of the other positive and all the negative clinical symptoms was also carried out using PANSS. The results showed that expect for hallucination, none of the other subscales of the PANSS were significantly correlated with the RSS. An additional file shows the correlations between the subscales on the PANSS and the RSS in all the participants with schizophrenia in more detail (see Additional file 1). In other words, in this study, among all the positive and negative subscales of PANSS, only hallucination was significantly correlated with RSS.

### Correlations between hallucination severity and GMV

**Fig. 3:**
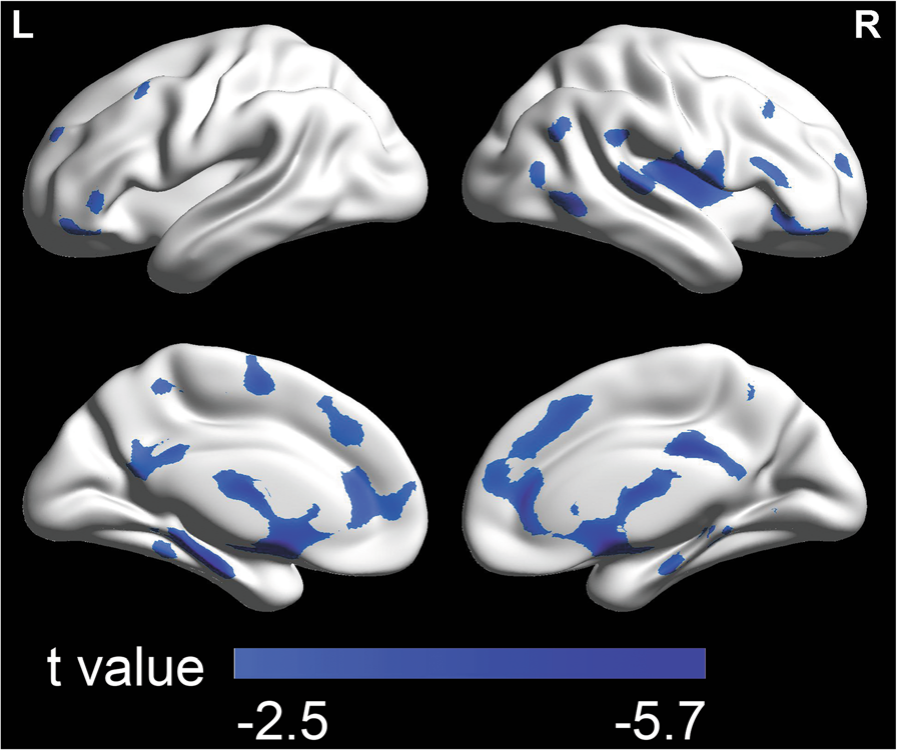
Brain areas with the gray matter volume (GMV) negatively associated with the hallucination severity in 33 participants with schizophrenia (*p* < 0.05, corrected, cluster size > 16,799 mm^3^). The colored bars show t values. L = left; R = right

In 33 participants with schizophrenia, the hallucination severity was significantly negatively correlated with the GMV in numeral brain regions (*p* < 0.05, corrected, cluster size > 16,799 mm3 (i.e., 16,799 voxels), see Table 2, Fig. 3), such as the left supplementary motor area (SMA) (BA 6), left superior frontal cortex (SFG) (BA 6/9), left precentral gyrus (BA 6), left parahippocampal gyrus (BA 30), right middle orbitofrontal cortex (BA 11), right inferior orbitofrontal cortex (BA 47), right rolandic operculum (BA 48), right insular cortex (BA 48), right superior temporal cortex (BA 22), bilateral middle frontal cortices (BA10/46), bilateral precuneus (BA 30), bilateral anterior cingulum cortices (BA 32), bilateral middle cingulum cortices (BA 24), bilateral hippocampus (BA 20), bilateral calcarine cortices (BA 17), and bilateral thalami. However, no significant correlations were revealed between the hallucination severity and the GMD in any brain region in participants with schizophrenia.

**Table 2:** Brain regions with GMV negatively correlated to hallucination severity in participants with schizophrenia

Number	Cluster size	Brain regions	Side	BA	MNI coordinate			Peak t value
					x	y	z	
1	102297	Superior frontal cortex	L	6	-24	-7	72	4.17
		Superior frontal cortex	L	9	-14	45	50	4.90
		Precentral gyrus	L	6	-24	-10	72	3.75
		Supplementary motor area	L	6	-4	-12	52	2.57
		Middle frontal cortex	L	10	-37	61	5	4.77
		Middle frontal cortex	L	46	-34	50	32	3.62
		Precuneus	L	30	-4	-54	10	2.71
		Precuneus	R	30	8	-52	8	2.75
		Anterior cingulum cortex	L	32	-3	44	13	3.11
		Anterior cingulum cortex	R	32	12	38	10	4.02
		Middle cingulum cortex	L	24	-3	28	37	2.88
		Middle cingulum cortex	R	24	6	27	34	2.97
		Calcarine cortex	L	17	-10	-58	11	2.82
		Calcarine cortex	R	17	4	-57	11	2.55
		Hippocampus	L	20	-24	-19	-13	4.82
		Hippocampus	R	20	24	-18	-16	3.17
		Parahippocampal gyrus	L	30	-18	-32	-12	4.26
		Thalamus	L	N.A	-5	-20	17	3.15
		Thalamus	R	N.A	7	-18	20	3.06
2	24376	Superior temporal cortex	R	22	67	-40	22	3.97
		Rolandic operculum	R	48	47	-4	6	3.35
		Insular cortex	R	48	40	-13	13	4.08
3	16800	Middle frontal cortex	R	10	38	59	8	3.26
		Middle frontal cortex	R	46	41	39	30	3.02
		Middle orbitofrontal cortex	R	11	18	67	-11	4.04
		Inferior orbitofrontal cortex	R	47	47	45	-13	3.93

*GMV* gray matter volume, *BA* Broadmann area, *MNI* Montreal Neurological Institute, *R* right, *L* left

Automated Anatomical Labeling (AAL) software [94] and the Brodmann templates implemented in MRIcroN software (https://www.nitrc.org/plugins/mwiki/index.php/mricron:MainPage) were employed to define Brodmann area

### Correlations between RSS and GMV

**Fig. 4:**
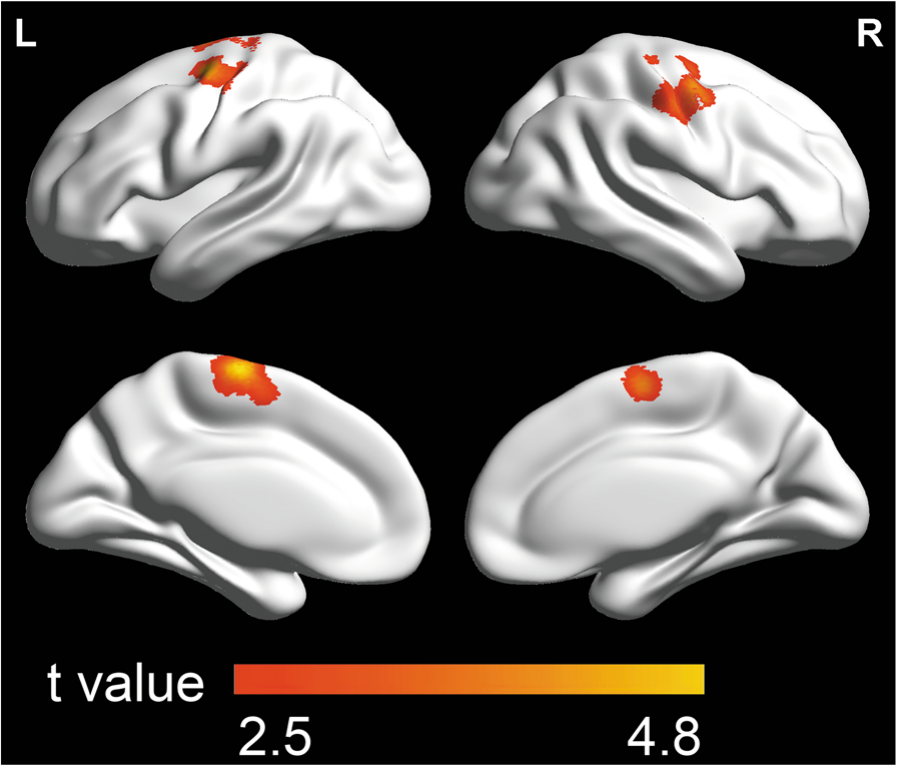
Brain areas with the gray matter volume (GMV) positively associated with the responsive search score (RSS) in 33 participants with schizophrenia (p < 0.05, corrected, cluster size > 15,141 mm^3^). The colored bars show t values. L = left; R = right

In 33 participants with schizophrenia, the RSS was significantly positively correlated with the GMV in the left SMA (BA6), left SFG (BA6), bilateral precentral gyri (BA 6), bilateral postcentral gyri (BA4), and bilateral middle frontal cortices (BA6) (*p* < 0.05, corrected, cluster size > 15,141 mm^3^, see Table 3, Fig. 4). There was no significantly negative correlation between the RSS and the GMV of any brain region in people with schizophrenia. No significant correlation could be revealed between the RSS and the GMV in any brain region in HCs.

**Table 3:** Brain regions with the GMV positively associated with the RSS in participants with schizophrenia

Number	Cluster size	Brain regions	Side	BA	MNI coordinate			Peak t value
					x	y	z	
1	37729	Superior frontal cortex	L	6	-26	-2	61	2.77
		Precentral gyrus	L	6	-34	-6	57	4.72
		Postcentral gyrus	L	4	-52	-15	51	3.37
		Supplementary motor area	L	6	-6	-13	64	4.53
		Middle frontal cortex	L	6	-26	1	63	2.70
2	15142	Precentral gyrus	R	6	54	0	43	3.63
		Postcentral gyrus	R	4	52	-8	38	2.83
		Middle frontal cortex	R	6	41	-6	55	3.40

*GMV* gray matter volume, *RSS* responsive search score, *BA* Broadmann area, *MNI* Montreal Neurological Institute, *R* right, *L* left

Automated Anatomical Labeling (AAL) software [94] and the Brodmann templates implemented in MRIcroN software (https://www.nitrc.org/plugins/mwiki/index.php/mricron:MainPage) were employed to define Brodmann area

### Overlapped mapping

**Fig. 5:**
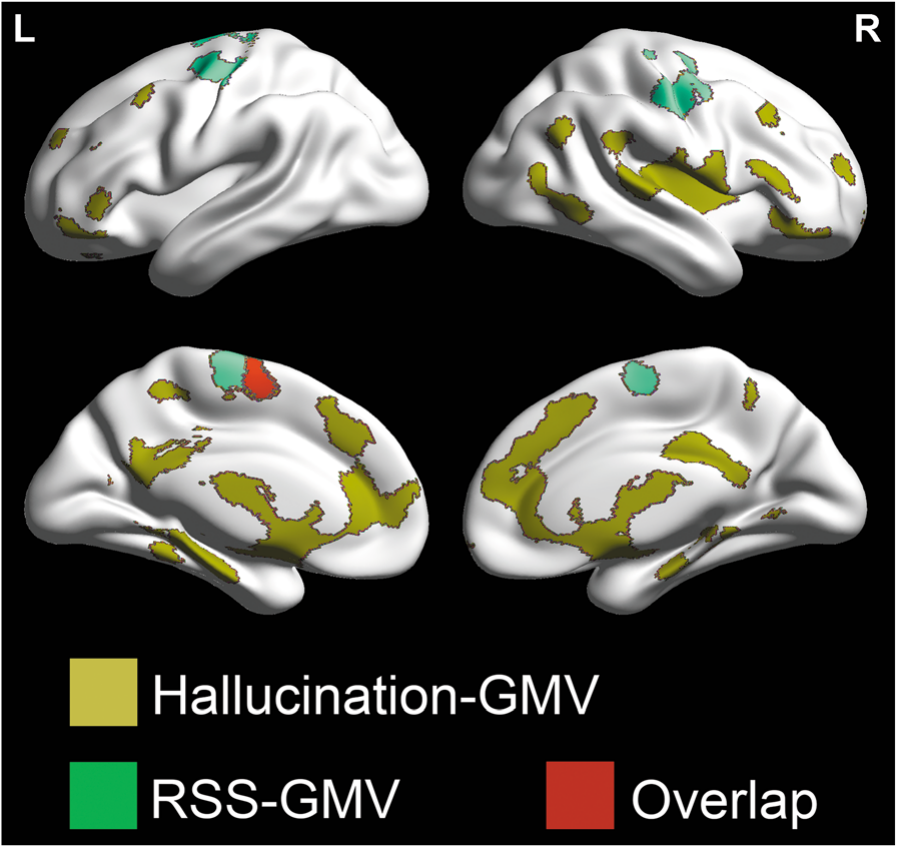
Overlapped areas (red color) between brain areas (deep yellow color) with GMV associated with the hallucination severity, and brain areas (green color) with the gray matter volume (GMV) associated with the responsive search score (RSS) in 33 participants with schizophrenia. L = left; R = right

In 33 participants with schizophrenia, the overlapped mapping revealed that the GMV of several brain regions was not only significantly negatively correlated with the hallucination severity but also significantly positively correlated with the RSS. These brain regions included the left SMA (BA6), left SFG (BA6), and left precentral gyrus (BA6) (Fig. 5).

## Discussion

### Negative correlation between hallucination severity and RSS

Studies of behavioral/cognitive correlates of positive and/or negative symptoms of schizophrenia are critical for understanding the pathopsychological processes underlying this mental disorder [3, 53, 54]. Although an uncorrected *p*-value was used, to our knowledge, this study firstly revealed that the RSS in participants with schizophrenia was significantly negatively correlated with the hallucination severity, but not significantly correlated with the other positive subscales and all the negative subscales on the PANSS. Thus, these results open a new research avenue on whether the RSS may be used as a potentially specific predictor of hallucination severity in people with schizophrenia, and whether RSS and hallucination in schizophrenia may share certain common underlying pathophysiological processes.

### Negative correlation between hallucination severity and GMV

It has been known that in people with schizophrenia the positive symptom subscale (including hallucinations) is significantly negatively correlated with not only the GMV in the frontal, parietal and temporal regions [44, 50, 52] but also that in the paralimbic system [39]. Particularly, the severity of hallucination is significantly negatively correlated with the GMV in the bilateral frontal, bilateral temporal, and left parietal cortices [51], and especially with the GMV in the left SFG [49]. The findings of the present study indicate that the hallucination severity is significantly negatively correlated with the GMV in numerous brain regions, such as the left SMA, left SFG, left precentral gyrus, left parahippocampal gyrus, right middle orbitofrontal cortex, right inferior orbitofrontal cortex, right rolandic operculum, right insular cortex, right superior temporal cortex, bilateral middle frontal cortices, bilateral precuneus, bilateral anterior cingulum cortices, bilateral middle cingulum cortices, bilateral hippocampus, bilateral calcarine cortices, and bilateral thalami. The results of this study support and extend the previous findings that the GMV in widespread cortical regions (including the frontal, temporal, and parietal cortices) is significantly negatively correlated with the hallucination severity [44, 49–52, 66].

However, in this study no significant correlation was revealed between the hallucination severity and the GMD in any brain regions. The results of this study are consistent with the previous reports that hallucinations are markedly associated with volumetric changes of certain brain structures [39, 44, 49–52].

Among these brain regions we are particularly interested in those whose GMV is not only significantly negatively correlated with hallucination, but also significantly positively with the RSS, including the left SMA, left SFG, and left precentral gyrus.

Some previous studies have already shown that the SMA is involved in auditory verbal hallucination (AVH) [67–70]. What are the potential mechanisms underlying the involvement of SMA in hallucinations? As mentioned above, the SMA plays a role in both awareness of intention to make a physical movement [71, 72] and mental imagery [69, 73], suggesting the impairment of the SMA function weakens the distinction between one’s own mental processing (awareness of intention to make a movement or auditory verbal imagery) and certain similar processing that is attributed to hallucinations, including AVH [73]. Moreover, using functional magnetic resonance imaging (fMRI), Linden et al. (2011) [69] have revealed that activation of the SMA is associated with either imagery or hallucination of voice hearing. More specifically, during AVH, activation of the SMA and that of the superior temporal areas occur simultaneously, but during active voice-hearing imagery, the onset of activation of the SMA precedes that of the superior temporal areas [69, 70]. Also, Raij et al. (2012) [73] have reported that hallucinations were associated with weakened activation of the SMA compared to imagery. Thus, the GMV-related impairments of the SMA functions, including the weakened awareness of intention to make a physical movement and declined mental imagery, may facilitate the occurrence of hallucinations.

The results of the present study also showed that the hallucination severity was significantly negatively correlated with the GMV in the left SFG, supporting the previous reports that the left SFG is involved in hallucinations [68, 74] and more in detail the GMV of the left SFG is significantly negatively correlated with severity of hallucination [68]. It has also been known that the left SFG plays a critical role in mediating working memory [75]. The recent Jenkins et al. study (2017) [76] has provided evidence showing that working memory can be used for predicting the presence of AVH in both people with schizophrenia and those with bipolar disorder. Thus, deficits in working memory may share some underlying mechanisms with the genesis of AVH and the involvement of the left SFG in hallucinations may be caused by the functional impairment of this cortical region in working memory.

In people with schizophrenia, the left precentral gyrus is involved in auditory verbal imagery [77] and among the brain regions whose activity is specifically related to AVH [70, 78]. In the future, it is of interest to investigate how functional connection between the SMA, SFG and precentral gyrus contributes to the induction of hallucinations in schizophrenia.

### Positive correlation between RSS and GMV

Previous studies have shown that there is a widely distributed reduction of GMV in people with schizophrenia in the left SMA, the left middle frontal gyrus, the left angular gyrus, the left superior temporal gyrus, the left cerebellar hemisphere, the right opercular area, and bilateral SFG [43]. In addition, the schizophrenia-related GMV reduction also extends to the anterior and posterior cingulate regions, amygdala, insula, superior temporal gyrus, thalamus, and parahippocampal gryus [45]. This present study also revealed that across participants with schizophrenia, the RSS was significantly positively correlated with the GMV in the left SFG, left SMA, bilateral precentral gyri, bilateral postcentral gyri, and bilateral middle frontal cortices. These findings support the view that regional abnormalities in certain brain structures, including the left SMA, left SFG, and left precentral gyrus, are associated with certain impaired cognitive domains in people with schizophrenia [79], thereby being important for approaching the potential pathophysiological processes underlying schizophrenia-related RSS deficits.

As mentioned in the Background, the RSS is based on the integration of several perceptual / cognitive processes, including fine discrimination, selective and sustained attention, perception, working memory, and active imagery. It is of importance to know whether some of these brain regions with the GMV positively correlated with RSS play a critical role in mediating the perceptual / cognitive / executive processes underlying the RSS. It has been known that in addition to the contribution of the SMA and precentral gyrus to eye-movement control [80], the SMA is involved in response selection operations in visual search tasks [81], perhaps through its functional connectivity with the posterior parietal cortex, which is the core cortical region for the visual search performance [82], especially when the cognitive demand becomes higher [83]. The SMA plays a role in both awareness of intention to make a physical movement [71, 72] and mental imagery [69, 73], which are also important to EEM. Moreover, the left SFG is also involved in both eye movement by its axonal connections with the cerebellum [84], particularly for smooth pursuit [85] and visual research [86]. The involvement of the left SFG in visual search is also closely associated with not only working memory [87, 88] but also preparation of executing movements including visual search [89]. This study revealed that the RSS was significantly positively correlated with the GMV in several brain regions, among which, the left SMA, right middle frontal cortex and bilateral precentral gyri in the occipito-tempro-frontal circuitry, which underlies both visual information and eye movement processing [12], also exhibited significantly positive correlations between their GMD and the RSS in participants with schizophrenia as revealed by Qiu et al., (2011) [12]. However, other brain regions in the occipito-tempro-frontal circuitry, such as the right inferior temporal cortex, right superior occipital cortex, and bilateral frontal eye fields, whose GMD was significantly positively correlated with the RSS [12], were not within the brain regions whose GMV was significantly positively correlated with the RSS in participants with schizophrenia in this study. Thus, the findings of this study suggest that in people with schizophrenia the vulnerability of RSS to changes of the GMV in the left SMA, left SFG, and left precentral gyrus which are considered as sensorimotor/motor executive cortical areas in the left hemisphere reflects the integrated functional changes in response selection operations, preparation of executing movement, active imagery, and working memory.

In this study, no significant correlation could be revealed between the RSS and the GMV of any brain regions in HCs. It is of interest and importance to know whether the occurrence of the positive correlation between the RSS and GMV in people with schizophrenia reflects the schizophrenia-related compensatory neural mechanisms underlying these perceptual/cognitive processes. The compensatory mechanism specifically associated with schizophrenia is an important issue [90, 91], and compensatory re-modelling processes may contribute to the cortical thickness variations in different stages of schizophrenia [92].

### The brain regions whose GMV was correlated with both hallucination severity and RSS

The most essential findings of this study shown by our overlapped mapping results are that the GMV of the SMA, SFG, and precentral gyrus in the left hemisphere is not only negatively correlated with the hallucination severity but also positively correlated with the RSS, suggesting that the schizophrenia-related GMV in these three cortical regions contribute to both the hallucination occurrence and the RSS.

The RSS reflects the integrated ability in both perceptual / cognitive processing and executive processing [12]. Thus, not only the SMA-damage-related impairments in either awareness of intention to make a physical movement or mental imagery, but also the SFG-damage-related impairment in either executive processing or working memory, can reduce the RSS.

Hallucinations, especially auditory hallucinations, are tightly associated with impaired executive processing [1]. As mentioned above, numerous studies have confirmed that the SMA, SFG, and precentral gyrus are involved in not only eye-movement control [12, 80, 84, 85, 93] but also AVH [67–70, 74, 78]. More specifically, the SMA and precentral gyrus particularly play a role in both awareness of intention to make a physical movement [71, 72] and mental imagery [69, 73, 77]. Since an impairment of the SMA function (such as the slowness of neural responses) weakens the distinction between one’s own mental processing (awareness of intention to make a movement or auditory verbal imagery) and the processing of hallucinations [73], a schizophrenia-related impairment of the GMV of the SMA may cause both deficits of RSS and induction of hallucinations. On the other hand, the GMV-related impairments of the SFG functions include the decline in working memory [75]. It has been shown that working memory is underlying the involvement of the left SFG in both visual search [87, 88] and the EEM that can be efficiently measured with RSS [12]. Since deficits in working memory share some underlying mechanisms with the genesis of AVH [76], the negative correlation between the hallucination severity and the GMV in the left SFG and positive correlation between the RSS score and the GMV of the left SFG suggest that schizophrenia-related hallucinations and RSS deficits may be associated with deficits in working memory.

In summary, the results of this study suggest that the GMV in the left SMA, left SFG, and left precentral gyrus are associated with both the hallucination incidence and RSS in schizophrenia. In the future, more investigation will be carried out to further examine whether the SMA, SFG and precentral gyrus are the three functionally connected complementary interfaces whose functional deficits caused by GMV alternations affects either RSS or hallucinations differently.

### Limitations

A few limitations in this study should be mentioned. First, the participants with schizophrenia had various illness durations, and they were all exposed to antipsychotic medications, which might have certain influences to the results. We have considered these influences and treated them in statistical analyses, however, they might still have some certain influences on cerebral cortical structures. Moreover, the present study only revealed GMV in a few brain structures associated with both the hallucination severity and RSS in people with schizophrenia. Due to the considerable heterogeneity in brain morphology, the modulation step in GMV measures might result in less consistent spatial localization of GMV differences from sample to sample. In the future, other estimations of brain morphology, such as the cortical thickness and gray matter mass (defined as GMD × GMV) of cortical regions, should be employed to further explore the neural substrates underlying the RSS and/or the generation of hallucination in people with schizophrenia.

## Conclusions

The hallucination severity is negatively correlated with the RSS of the EEM in people with schizophrenia. Moreover, the GMV of the SMA, SFG, and precentral gyrus in the left hemisphere is negatively correlated with the hallucination severity and positively correlated with the RSS in people with schizophrenia. These findings demonstrate an association between the hallucination severity and the RSS. The GMV of the left SMA, left SFG and left precentral gyrus may be the common substrates underlying the hallucination inductions and RSS in schizophrenia. Thus, in the future it is important to further investigate whether the RSS can be used as a reliable biomarker for predicting the hallucination severity of schizophrenia. This line of investigation can not only help reveal the underlying pathophysiological mechanisms of schizophrenia but also determine whether the RSS is an endophenotype for schizophrenia.

## Additional file

Additional file 1: Correlations between the subscales on the PANSS and the RSS in 33 participants with schizophrenia. (DOCX 27 kb)

## Abbreviations

AVH: Auditory verbal hallucination
BA: Broadmann area
EEM: Exploratory eye movement
FWHM: Full width-half maximum
GMD: Gray matter density
GMV: Gray matter volume
HCs: Healthy controls
HMRF: Hidden Markov Random Field
L: left
MESL: Mean eye scanning length
MNI: Montreal Neurological Institute
MRI: Magnetic resonance imaging
NEFs: Number of eye fixations
PANSS: Positive and Negative Syndrome Scale
R: Right
RSS: Responsive search score
SFG: Superior frontal cortex
SMA: Supplementary motor area
TESL: Total eye scanning length
