# Supplementary material for: Correlations between exploratory eye movement, hallucination, and cortical gray matter volume in people with schizophrenia
Source: BMC Psychiatry. 2018 Jul 13;18:226. doi: 10.1186/s12888-018-1806-8 (PMC6045825; doi:10.1186/s12888-018-1806-8)
Supplement: Supplementary file 1 — Correlations between the subscales on the PANSS and the RSS in 33 participants with schizophrenia. (DOCX 27 kb) [file 12888_2018_1806_MOESM1_ESM.docx]

**Additional file 1** Correlations between the subscales on the PANSS and the RSS in 33 participants with schizophrenia

| Subscales on PANSS | P value |
| --- | --- |
| Positive scale |  |
| Delusions | 0.744 |
| Conceptual disorganization  Hallucinations  Hyperactivity  Grandiosity  Suspiciousness/persecution  Hostility  Negative scale  Blunted affect  Emotional withdrawal  Poor rapport  Passive/apathetic social withdrawal  Difficulty in abstract thinking  Lack of spontaneity and flow of conversation  Stereotyped thinking | 0.139  0.043  0.053  0.084  0.335  0.732  0.311  0.520  0.310  0.959  0.346  0.341  0.794 |

PANSS Positive and Negative Syndrome Scale; RSS responsive search score

Spearman's correlation (p < 0.05, uncorrected)
